# Antimicrobial resistance of *Escherichia coli*, *Enterobacter* spp., *Klebsiella pneumoniae* and *Enterococcus* spp. isolated from the feces of giant panda

**DOI:** 10.1186/s12866-022-02514-0

**Published:** 2022-04-14

**Authors:** Xin Wang, Yi Zhang, Caiwu Li, Guo Li, Daifu Wu, Ti Li, Yuanyuan Qu, Wenwen Deng, Yongguo He, Petri Penttinen, Hemin Zhang, Yan Huang, Ke Zhao, Likou Zou

**Affiliations:** 1 College of Resources, Sichuan Agricultural University, Chengdu, Sichuan China; 2 College of Life Sciences, Zhejiang University, Zhejiang, Hangzhou China; 3 Key Laboratory of SFGA on Conservation Biology of Rare Animals in the Giant Panda National Park, China Conservation and Research Center for Giant Panda, Dujiangyan, Sichuan China

**Keywords:** Antimicrobial resistance, Resistance genes, Whole-genome sequencing, Giant panda

## Abstract

**Background:**

*Escherichia coli*, *Enterobacter* spp., *Klebsiella pneumoniae* and *Enterococcus* spp., common gut bacteria in giant pandas, include opportunistic pathogens. The giant panda is an endangered species, classified as vulnerable by the World Wildlife Foundation. Continuous monitoring for the emergence of antimicrobial resistance (AMR) among bacterial isolates from giant pandas is vital not only for their protection but also for public health.

**Results:**

A total of 166 *E. coli*, 68 *Enterobacter* spp.*,* 116 *K. pneumoniae* and 117 *Enterococcus* spp. isolates were collected from fecal samples of 166 giant pandas. In the antimicrobial susceptibility tests, 144 *E. coli* isolates, 66 *Enterobacter* spp. isolates, 110 *K. pneumoniae* isolates and 43 *Enterococcus* spp. isolates were resistant to at least one antimicrobial. The resistant isolates carried antimicrobial resistance genes (ARGs), including *sul3*, *bla*_TEM_, *bla*_SHV_ and *tetA*. The differences in the prevalence of the *bla* types implied that the genetic basis for β-lactam resistance among the *E. coli, Enterobacter* spp. and *K. pneumoniae* isolates was different. The strain *K. pneumoniae* K85 that was resistant to sixteen antimicrobials was selected for whole genome sequencing. The genome contained Col440I, IncFIB_K_ and IncFII_K_ plasmids and altogether 258 ARGs were predicted in the genome; 179 of the predicted ARGs were efflux pump genes. The genetic environment of the β-lactamase genes *bla*_CTX-M-3_ and *bla*_TEM-1_ in the *K. pneumoniae* K85 genome was relatively similar to those in other sequenced *K. pneumoniae* genomes. In comparing the giant panda age groups, the differences in the resistance rates among *E. coli*, *K. pneumoniae* and *Enterobacter* spp. isolates suggested that the infections in giant pandas of different age should be treated differently.

**Conclusions:**

Antimicrobial resistance was prevalent in the bacterial isolates from the giant pandas, implying that the gut bacteria may pose serious health risks for captive giant pandas. The resistance genes in the genome of *K. pneumoniae* K85 were associated with insertion sequences and integron-integrase genes, implying a potential for the further spread of the antimicrobial resistance.

**Supplementary Information:**

The online version contains supplementary material available at 10.1186/s12866-022-02514-0.

## Background

The giant panda, *Ailuropoda melanoleuca*, is a mammal species endemic to China, where the sparse giant panda population is limited to Sichuan, Shanxi and Gansu provinces [1]. The captive panda population was approximately 600 by the end of 2019. Although the number of both wild and captive pandas has increased, the giant pandas are still endangered due to several threats. Intestinal tract diseases caused by pathogenic bacteria has become a considerable threat to the health of giant pandas [2]. *Escherichia coli*, *Enterobacter* spp., *Klebsiella* and *Enterococcus* spp. are common gut bacteria in humans and other animals, including giant pandas [3–5]. These species play important commensal roles in gut; however, they are also opportunistic pathogens, and can cause various diseases [6–8]. For example, some *E. coli* strains cause hemorrhagic colitis, and these enterohaemorrhagic *E. coli* have been isolated from giant pandas [9]. *Enterobacter* spp., *K. pneumoniae* and *Enterococcus faecium* have been associated with hospital-acquired infections and outbreaks [10–15]. Clinical infections caused by *Enterococcus* spp. have been increasing in recent years [16], and *Klebsiella* and *Enterobacter* spp. can cause a wide range of infections [12, 17–19].

Antimicrobials have been widely used to prevent and cure infectious diseases in captive giant pandas in recent decades [20–22]. However, with the widespread use of antimicrobials, the number of drug-resistant strains has increased and the development and spread of multidrug-resistant (MDR) bacteria in humans and the environment has accelerated [23]. In China, more antimicrobial agents are consumed than in most other countries. According to a 2007 survey, almost half of the 210,000 tons of antimicrobials produced in China were used in livestock as therapeutic drugs and feed additives [24]. In addition, antimicrobials like ceftriaxone sodium are used not only in humans but also in giant pandas [25]. Thus, antimicrobial resistant strains may develop in giant pandas and spread to humans and other animals.

Antimicrobial resistance has caused serious problems in clinical practice [26]. Infections by *Klebsiella* spp., especially *K. pneumonia*, are frequently caused by MDR strains that produce extended-spectrum β-lactamases (ESBLs; mainly including *bla*_TEM_, *bla*_CTX-M_, *bla*_SHV_ and *bla*_GES_ types) [19, 26]. *K. pneumonia* may be also naturally resistant to certain antimicrobials, including ampicillin, amoxicillin, carbenicillin and ticarcillin [27, 28]. Likewise, *Enterobacter* spp., especially *Enterobacter cloacae*, may be naturally resistant to, for example, ampicillin, kanamycin and tetracycline [7]. Generally, *Enterococcus* spp. are intrinsically resistant to many antimicrobials and can easily acquire resistance to other agents [29]. Acquired high-level aminoglycoside or penicillin resistance, as well as erythromycin or tetracycline resistance, have increased among *Enterococcus* spp. [16, 30, 31].

Several investigations have been carried out to monitor the distribution of antimicrobials and disinfectant resistance genes in *E. coli* and *K. pneumoniae* isolates from the giant pandas [2, 20, 22, 32]. In giant pandas, *E. coli* infections were frequently caused by MDR strains [2, 20, 32]. To our knowledge, detailed gene and genome level information on the antimicrobial resistant bacteria, especially on *Enterobacter* and *Enterococcus* spp., from giant pandas is still lacking. Therefore, comprehensive investigation at molecular level to monitor the distribution of antimicrobial resistant, opportunistic pathogens from giant pandas was needed. We isolated *E. coli, Enterobacter* spp., *K. pneumoniae* and *Enterococcus* spp. from giant panda feces and assessed their antimicrobial resistance and related genetic properties, with the aims to 1) characterize the antimicrobial resistance phenotypes and genotypes, 2) compare the antimicrobial resistance between the four taxa, and to 3) further understand the resistance based on whole-genome sequencing of a MDR *K. pneumoniae* isolate.

## Results

### Antimicrobial susceptibility of all isolates

**Fig. 1:**
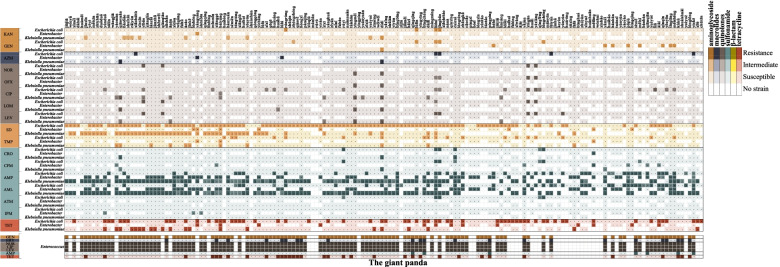
Antimicrobial resistance of *E. coli*, *Enterobacter* spp.,* K. pneumoniae* and *Enterococcus* spp. isolates against 18 antimicrobials. The indicator on the right denotes the relationship between the antimicrobial resistance and color range. KAN, kanamycin; GEN, gentamicin; AZM, azithromycin; ERY, erythromycin; NOR, norfloxacin; OFX, ofloxacin; CIP, ciprofloxacin; LOM, lomefloxacin; LEV, levofloxacin; SD, sulfadiazine; TMP, trimethoprim; CRO, ceftriaxone; CFX, cefixime; AMP, ampicillin; AML, amoxicillin; ATM, aztreonam; IPM, imipenem; TET, tetracycline

A total of 166 *E. coli*, 68 *Enterobacter* spp.*,* 116 *K. pneumoniae* and 117 *Enterococcus* spp. isolates were purified from fecal samples of 166 giant pandas. Only one isolate per genus per giant panda was kept for further analyses. In the antimicrobial susceptibility tests, 87% (*n* = 144) *E. coli* isolates, 97% (*n* = 68) *Enterobacter* spp. isolates, 95% (*n* = 110) *K. pneumoniae* isolates and 37% (*n* = 37) *Enterococcus* spp. isolates were resistant to at least one antimicrobial (Fig. 1, Supplementary Table S1).

**Fig. 2:**
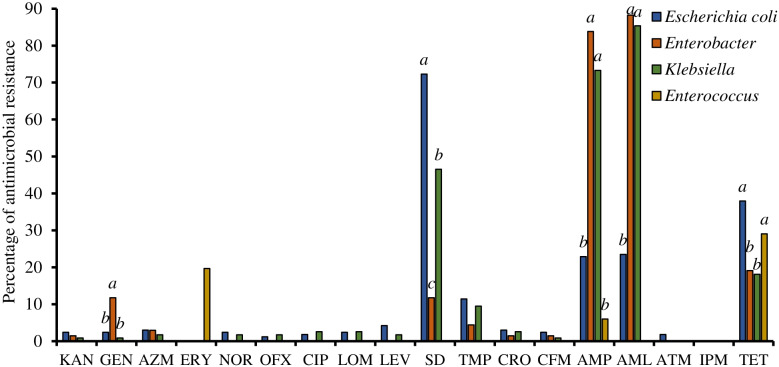
Antimicrobial resistance of *E. coli*, *Enterobacter* spp., *K. pneumoniae* and *Enterococcus* spp. isolates against 18 antimicrobial agents. KAN, kanamycin; GEN, gentamicin; AZM, azithromycin; ERY, erythromycin; NOR, norfloxacin; OFX, ofloxacin; CIP, ciprofloxacin; LOM, lomefloxacin; LEV, levofloxacin; SD, sulfadiazine; TMP, trimethoprim; CRO, ceftriaxone; CFX, cefixime; AMP, ampicillin; AML, amoxicillin; ATM, aztreonam; IPM, imipenem; TET, tetracycline. Different letters above columns indicate statistically significant differences at *P* < 0.05

Many of the isolates were resistant to at least three different antimicrobial classes and were considered MDR strains. Out of the *E. coli* isolates, 72% were resistant to sulfadiazine (SD), 38% to tetracycline (TET), and approximately 23% to amoxicillin (AML) and ampicillin (AMP) (Fig. 2); 18% (*n* = 29) were resistant to three or more tested antimicrobials (Supplementary Table S1). Out of the *Enterobacter* spp. isolates, 88% were resistant to AML, 84% to AMP, and 7% (*n* = 5) were MDR strains. Out of the *K. pneumoniae* isolates, 85% were resistant to AML, 73% to AMP, 47% to SD, and 15% (*n* = 17) were MDR strains. Out of the *Enterococcus* spp. isolates, 35% were resistant to TET, 29% to erythromycin (ERY), and 3% (*n* = 15) were MDR strains.

The prevalence of gentamicin (GEN) resistance was highest among the *Enterobacter* spp. isolates, and that of SD resistance was highest among the *E. coli* isolates and second highest among the *K. pneumoniae* isolates. The prevalence of AMP and AML resistances were highest and that of TET lowest among the *Enterobacter* spp. and *K. pneumoniae* isolates*.*

### Antimicrobial resistant strains by giant panda sex and age

Antimicrobial resistant isolates were detected in 161 of the 166 giant pandas (Fig. 1). The difference in the proportion of antimicrobial resistant isolates from female and male giant pandas was limited to *Enterococcus* isolates: 26.9 and 46.0% of the isolates from females and males, respectively, were resistant to tetracycline (*P* < 0.05) (Fig. S1).

**Fig. 3:**
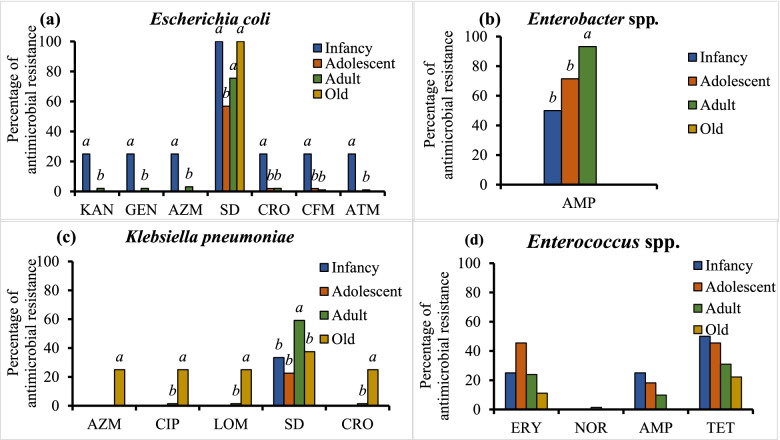
The proportion of antimicrobial resistant isolates from (**a**) infant, (**b**) adolescent, (**c**) adult and (**d**) old giant pandas. KAN, kanamycin; GEN, gentamicin; AZM, azithromycin; ERY, erythromycin; NOR, norfloxacin; OFX, ofloxacin; CIP, ciprofloxacin; LOM, lomefloxacin; LEV, levofloxacin; SD, sulfadiazine; TMP, trimethoprim; CRO, ceftriaxone; CFX, cefixime; AMP, ampicillin; AML, amoxicillin; ATM, aztreonam; IPM, imipenem; TET, tetracycline

Among the *E. coli* isolates, the prevalence of resistance to six antimicrobials was highest in isolates from pandas in their infancy (*P* < 0.05) (Fig. 3a). All the *E. coli* isolates from infant and old pandas were resistant to SD, and the prevalence of SD resistance was lowest among isolates from adolescent pandas (*P* < 0.05). The prevalence of AMP resistance was higher among *Enterobacter* spp. isolates from adult pandas than among those from infant and adolescent pandas (*P* < 0.05) (Fig. 3b). For the *K. pneumoniae* isolates, the prevalence of resistance to four antimicrobials was highest in isolates from old pandas (*P* < 0.05), and the prevalence of SD resistance was highest in isolates from adult pandas (*P* < 0.05) (Fig. 3c). For *Enterococcus* spp. isolates, there was almost no significant difference in resistance to tested antimicrobials among giant pandas of different ages (Fig. 3d).

### Prevalence of ARGs

The genotypes of antimicrobial resistant *E. coli*, *Enterobacter* spp., *K. pneumoniae* and *Enterococcus* spp. isolates were characterized by analyzing the ARGs in the isolates with different antimicrobial resistance phenotypes.

Among the *E. coli* isolates, *tetA* was detected in 67% (42/63) of the tetracycline-resistant isolates, *bla*_TEM_ and *bla*_CTX_ were detected in 36% (16/45) and 18% (8/45) of the β-lactam-resistant isolates, respectively, *sul2* and *sul3* were detected in 7 and 9% of the sulfonamide-resistant isolates, respectively, *qnrB* was detected in one of the eight fluoroquinolone-resistant isolates, and both *acc (3)-IIa* and *ant (3″)-Ia* were detected in one of the five aminoglycoside-resistant isolates (Table 1).

**Table 1:** Resistance genes and genetic elements in antimicrobial resistant *E. coli*, *Enterobacter* spp., *K. pneumoniae* and *Enterococcus* spp. isolates from the feces of giant pandas in China

Resistance phenotype	Resistance gene	Number of resistance genes or genetic elements / Number of antimicrobial-resistant isolates			
		*E. coli*	*Enterobacter*	*K. pneumoniae*	*Enterococcus*
Aminoglycosides	*acc (3)-IIa*	1/5	0/9	0/1	–
	*aph (3′)-Iia*	0/5	0/9	0/1	–
	*acc (6′)-Ib*	0/5	0/9	1/1	–
	*ant (3″)-Ia*	1/5	1/9	0/1	–
Macrolides	*ermE*	–	–	–	8/34
Quinolones	*qnrA*	0/8	–	0/3	–
	*qnrB*	1/8	–	1/3	–
Sulfonamides	*sul1*	1/121	0/8	7/54	–
	*sul2*	9/121	2/8	4/54	–
	*sul3*	11/121	0/8	3/54	–
β-Lactams	*bla*_TEM_	16/45	4/62	7/99	–
	*bla*_VIM_	0/45	0/62	2/99	–
	*bla*_SHV_	1/45	7/62	82/99	–
	*bla*_CTX_	8/45	5/62	8/99	–
	*bla*_IPM_	0/45	0/62	0/99	–
Tetracyclines	*tetA*	42/63	10/13	16/21	–
	*tetB*	2/63	1/13	0/21	–
	*tetC*	2/63	0/13	2/21	–
	*tetM*	–	–	–	8/23
	*tetL*	–	–	–	8/23

-, resistance gene not detected

The gene *tetA* was detected in 77% (10/13) of the tetracycline-resistant *Enterobacter* spp. isolates, *bla*_TEM_, *bla*_SHV_ and *bla*_CTX_ were detected in 11% or less of the β-lactam-resistant isolates, *sul1* was detected in two of the eight sulfonamide-resistant isolates, and *ant (3″)-Ia* was detected in one of the nine aminoglycoside-resistant isolates (Table 1).

ARGs for β-lactam-resistance were detected in all the resistant *K. pneumoniae* isolates, with *bla*_SHV_ in 83% (82/99) of them, *tetA* was detected in 76% (10/13) of the tetracycline-resistant isolates, and *sul1*, *sul2* and *sul3* were detected in 13% or less of the sulfonamide-resistant isolates. The only *K. pneumoniae* aminoglycoside-resistant isolate carried *acc (6′)-Ib* gene.

The gene *ermE* was detected in 24% of the macrolide resistant *Enterococcus* spp. isolates, and 35% of the tetracycline resistant isolates carried *tetM* or *tetL* genes.

### Antibiotic resistance features in the *K. pneumoniae* K85 genome

The strain *K. pneumoniae* K85 that was resistant to sixteen antimicrobials was selected for whole genome sequencing. The 1454 reads (Clean Data) were assembled into 91 contigs with a combined length of 5,514,535 bp. The longest contig was 368,946 bp. A total of 5349 ORFs were detected in the *K. pneumoniae* K85 genome with an average gene length of 897 bp. *K. pneumoniae* K85 contained Col440I, IncFIB_K_ and IncFII_K_ plasmids. Altogether 258 ARGs were predicted in the *K. pneumoniae* K85 genome (Table 2). Altogether 179 of the predicted ARGs were efflux pump genes, and the rest were related to enzymatic inactivation of antimicrobials, alteration, protection and replacement of the antimicrobial target, and reduced permeability to antimicrobials. The predicted aminoglycoside-modifying enzyme genes included *aadA16*, *aph (3′)-Ia,* and *acc (6′)-Ib-cr* that can simultaneously confer fluoroquinolone resistance. The predicted *bla*_CTX-M-3_, *bla*_SHV-93_ and *bla*_TEM-1_ confer resistance to β-lactams. In addition, genes encoding general mechanisms that mediate antibiotic resistance to fluoroquinolone (*qnrB2* and *qnrS1*), sulfonamide (*sul1* and *sul3*) and tetracycline (*tet34* and *tetT*) were also predicted.

**Table 2:** Antimicrobial resistance genes in the genome of *K. pneumoniae* K85

Mechanism of antibiotic resistance	Resistance genes	Number of genes
Antibiotic efflux	*arlR*; *baeR*; *kdpE*; *adeR*; *Escherichia coli CpxR*; *facT*; *emrB*; *cpxA*; *leuO*; *bcr-1*; *bcrA*; *emrD*; *floR*; *hmrM*; *hp1181*; *lmrB*; *lrfA*; *macA*; *macB*; *mdfA*; *mdtG*; *mdtH*; *mdtK*; *mdtL*; *mdtM*; *mdtN*; *mdtO*; *mdtP*; *mexJ*; *msbA*; *msrB*; *norB*; *oleC*; *patA*; *patB*; *rosA*; *rosB*; *salA*; *sav1866*; *taeA*; *tcmA*; *tet(A)*; *tetA(48)*; *tetA(60)*; *tetB(60)*; *tolC*; *vgaB*; *yojI*; *Enterobacter cloacae rob*; *Escherichia coli rob*; *marA*; *ramA*; *acrE*; *adeF*; *adeL*; *adeS*; *baeS*; *cmeB*; *crp*; *emrA*; *emrK*; *emrR*; *emrY*; *Escherichia coli acrA*; *evgA*; *evgS*; *H-NS*; *mdtA*; *mdtF*; *mexA*; *mexK*; *muxB*; *oprM*; *oprZ*; *oqxA*; *sdiA*; *smeC*; *tcr3*	179
Antibiotic inactivation	*aadA16*; *aph (3′)-Ia*; *acc (6′)-Ib-cr*; *bla*_CTX-M-3_; *bla*_SHV-93_; *bla*_TEM-1_; *nmcR*; *fosA5*; *mphA*; *mrx*; *cmlv*; *arr-3*; *iri*; *rphB*	19
Antibiotic inactivation, antibiotic target alteration	*tet34*	1
Antibiotic target alteration	*vanRF*; *vanRE*; *vanRM*; *vanG*; *vanHB*; *gyrB*; *parY*; EF-Tu; *gyrA*; *UhpT*; *murA*; *kasA*; *katG*; *ndh*; *ileS*; *cls*; *acrS*; *soxR*; *clbB*; *rlmA (II)*; *arnA*; *eptB*; *pmrE*; *pmrF*; *bacA*	30
Antibiotic target alteration, antibiotic efflux	*basR*; *basS*	2
Antibiotic target protection	*mfd*; *qnrB2*; *qnrS1*; *tetT*; *vanRI*; *vanHD*; *vanTC*; *vanTN*; *pmrC*	12
Antibiotic target replacement	*mecC*; *dfrA3*; *dfrA5*; *dfrE*; *sul1*; *sul3*	8
Reduced permeability to antibiotic, resistance by absence	*E. coli LamB*	2
Reduced permeability to antibiotic	*K. pneumoniae* OmpK37	5

**Fig. 4:**
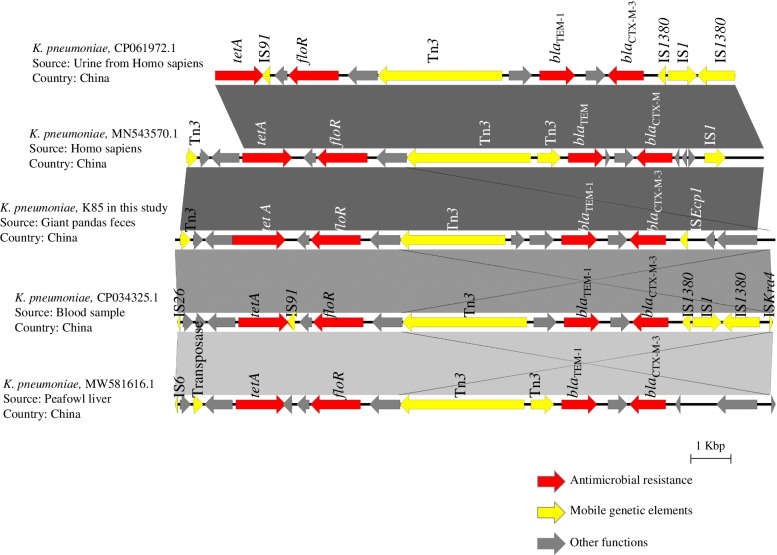
The genetic environments of *bla*_CTX-M-3_ and *bla*_TEM-1_ in the genome of *K. pneumoniae* K85 and reference genomes. Genes encoding antimicrobial resistance are indicated with red and other functions with grey, and mobile genetic elements with yellow

**Fig. 5:**
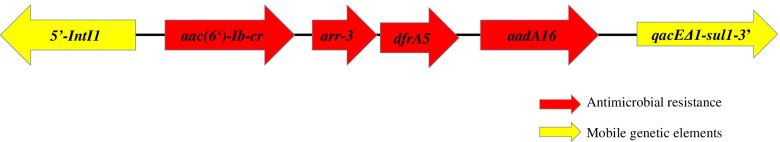
The structure of the class 1 integron resistance gene cassette in the genome of *K. pneumoniae* K85. Genes encoding antimicrobial resistance are indicated with red and mobile genetic elements with yellow

The genetic environment of the β-lactamase genes *bla*_CTX-M-3_ and *bla*_TEM-1_ in the *K. pneumoniae* K85 genome was relatively similar to those in other sequenced *K. pneumoniae* genomes (Fig. 4). The gene *bla*_TEM-1_ was adjacent to *bla*_CTX-M-3_, and this resistance region also included another two ARGs (*floR* and *tetA*) conferring resistance to tetracycline and florfenicol. More importantly, these antimicrobial resistance regions were flanked by various IS elements. The gene *bla*_TEM-1_ was adjacent to *bla*_CTX-M-3_, and this region also included *floR* and *tetA* that confer resistance to florfenicol and tetracycline, respectively. The isolate *K. pneumoniae* K85 harbored a class 1 integron gene cassette with resistance genes *aac (6′)-Ib-cr*, *arr-3*, *dfrA5* and *aadA16* (Fig. 5).

## Discussion

We studied the distribution of antimicrobial resistant, opportunistic pathogens in giant panda guts by isolating *E. coli, Enterobacter* spp., *K. pneumoniae* and *Enterococcus* spp. from giant panda feces. The results showed that antimicrobial resistance was common among the isolates, ranging from 95% or more among the *Enterobacter* spp. and *K. pneumoniae* isolates to 37% among the *Enterococcus* spp. isolates.

Our results showed that five *E. coli* isolates were resistant to ten or more antimicrobials, implying that MDR *E. coli* may pose serious health risks for captive giant pandas. Compared to the 88 *E. coli* strains from giant pandas in Bifengxia, China [20], in our study the antimicrobial resistance range of the isolates was wider and the prevalence of resistance to amoxicillin was higher. However, the prevalence of resistances to six antimicrobials were lower than an earlier study on giant pandas from Wolong and Dujiangyan, the China Conservation and Research Center for Giant Panda [22], possibly partly due to the controlled use of antimicrobials [32]. In addition, the variation in antimicrobial resistance profiles at different times and sites may result from giant pandas obtaining antimicrobial-resistant bacteria via contacts with feeders, feeding environment or tourists that violate the feeding regulations of the zoos [32–34], thus increasing the risks of cross-infection and exposure to pathogens and ARGs through the digestive tract.

*Enterobacter* spp. that are opportunistic pathogens in humans, fish and other animals [35–38] have been found in the intestines of giant pandas [8, 39]. However, to our knowledge their resistance to antimicrobials has not been investigated. Compared to our *E. coli* isolates, the rates of resistance to ampicillin and amoxicillin were higher among the *Enterobacter* spp. isolates. *Enterobacter* spp. carry resistance genes that promote the MDR phenotype [40–43], it could be due to their ability to acquire numerous genetic mobile elements containing resistance genes [44], making them a potential problem for giant pandas. Unlike the *Enterobacter* spp. strains from humans and companion animals [45, 46], the giant panda *Enterobacter* spp. isolates were not resistant to ciprofloxacin, indicating that quinolone antimicrobials may remain effective in treating *Enterobacter* infections [47].

Over 70% of the *K. pneumoniae* isolates were resistant to ampicillin and amoxicillin. The prevalence of ESBL-producing *K. pneumoniae* in many areas of the world has reached 50%, indicating that its antimicrobial resistance is ubiquitous [48]. In Asia, the prevalence of resistance to most of the commonly used antimicrobials is high among *K. pneumoniae* [49]. In China, the probabilities of MDR *K. pneumoniae* infections are high, so management of antimicrobial resistance in MDR *K. pneumoniae* has been a major challenge for clinical veterinarians. *K. pneumoniae* may play a key role in disseminating ARGs from environmental microbes to clinically important pathogens because of its wider ecological distribution, greater ARG diversity or a higher mobile genetic element burden than other Gram-negative opportunists [50, 51].

Studies on the antimicrobial resistance of *Enterococcus* spp. derived from giant pandas are few. In our study, the *Enterococcus* isolates were mainly resistant to tetracycline, erythromycin and ampicillin. Compared with the giant pandas, the rate of tetracycline resistance among wild rabbit-derived *Enterococcus* spp. was higher [52], possibly due to the contamination of water or vegetation in the woodlands by fecal material from wild birds or even humans [53]. The intrinsic resistance of *Enterococcus* spp. to semisynthetic penicillin, aminoglycosides, vancomycin, polymyxins and streptogramins has compromised the choice of therapeutic options for the treatment of enterococcal infections [54]. It is suggested that when treating *Enterococcus* infections, antimicrobials should be selected according to the susceptibility and resistance among the isolates to reduce the generation of antimicrobial-resistant strains and the spread of antimicrobial-resistance genes.

The only difference between the isolates from female and male giant pandas was the lower TET resistance rate in *Enterococcus* isolates from females*.* In comparing the giant panda age groups, the differences in the resistance rates among *E. coli*, *K. pneumoniae* and *Enterobacter* spp. isolates suggested that the infections in giant pandas of different age should be treated differently. Diet conversion from infancy to adolescence may induce higher prevalence of gastroenteritis that is treated with antimicrobials causing high antimicrobials-resistance rate [55]. In our study, the resistance prevalence to some antimicrobials were higher among the isolates from the infant giant pandas or the old giant pandas than in the other age groups. At the age of 7–18 months, the diet of the giant pandas changes gradually from breast milk or artificial milk to bamboo, which can lead to intestinal diseases and affect the health of the pandas [56]. The probability of intestinal infection is higher at old age because of weakened immunity, basic diseases and long-time application of wide-spectrum antimicrobials [20]. For the *K. pneumoniae* and *E. coli* isolates, the prevalence of resistance to sulfadiazine was highest and lowest, respectively, among isolates from adult pandas. The difference may be associated with differences in resistance mechanisms, spread of resistance genes or in inherent characteristics of the taxa, yet further research is needed to confirm the cause.

*Enterobacter* isolates are able to produce extended-spectrum β-lactamases of _CTX-M_, _TEM_ and _SHV_ types, and β-lactamases are the prominent reason for β-lactam resistance in most *Enterobacter* species [44]. The *bla*_TEM_, *bla*_SHV_ and *bla*_CTX-M_ genes that have been found in *Enterobacter* spp. isolates from other animals, including humans [57, 58], were detected in the isolates from the giant pandas as well. The differences in the prevalence of the *bla* types implied that the genetic basis for β-lactam resistance among the *E. coli, Enterobacter* spp. and *K. pneumoniae* isolates were different.

The genome of *K. pneumoniae* K85, an isolate resistant to sixteen antimicrobials, contained multiple ARGs. Efflux pump genes were the most numerous ARGs, indicating that the efflux pumps are the main determinants for the resistance. Efflux pumps are commonly found in bacteria and mediate resistance to antimicrobials, disinfectants, detergents and dyes [59]. Overexpression of the efflux pump genes can lead to multi-drug resistance: the efflux pump encoded by *emrE* can pump tetracycline, erythromycin, crystal violet and the stain ethidium bromide [60], and the pump encoded by mdfA can pump ciprofloxacin, kanamycin, neomycin, and quaternary ammonium disinfectants out of cells [61]. Even though *K. pneumoniae* K85 was resistant to all β-lactams except aztreonam, the genome of *K. pneumoniae* K85 contained the resistance gene *bla*_CTX-M-3_ that encodes an aztreonam hydrolyzing enzyme [62]. In addition, we detected mobile genetic elements including insertion sequences, transposons, integrons and plasmids that can mobilize antimicrobial resistance genes. The insertion sequence ISE*cp1*, adjacent to the *bla* genes in the K85 genome, is associated with the expression and mobilization of *bla*_CTX-M_ genes [63, 64]. Thus, the location of insertion sequences and integron-integrase genes next to the resistance genes in the genome of *K. pneumoniae* K85 implied a potential for gene transfer between different plasmids.

## Conclusions

In summary, the *E. coli*, *Enterobacter* spp., *K. pneumoniae* and *Enterococcus* spp. isolated from the feces of giant pandas showed resistance to various antimicrobials and carried several ARGs, implying that the gut bacteria may pose serious health risks for captive giant pandas. The resistance genes in the genome of *K. pneumoniae* K85 were associated with insertion sequences and integron-integrase genes, implying a potential for the further spread of the antimicrobial resistance.

## Materials and methods

### Bacterial isolation and identification

Fresh feces of 166 giant pandas were sampled in May to June 2018, including eight infant giant pandas (aged < 1.5 year), 51 adolescent giant pandas (aged 1.6 to 5 years), 98 adult giant pandas (aged 6 to 20 years) and nine old giant pandas (aged > 21 years) (Supplementary Table S2). Twenty-five-gram samples were taken aseptically, placed in sterile conical flasks with 225 mL of buffered peptone water (BPW; Huankai Microbial Technology Co., Ltd., Guangzhou, China) and incubated for 16–18 h at 200 rpm at room temperature. One loopful of overnight BPW culture was streaked onto MacConkey agar (MAC) and eosin methylene blue agar (EMB), Simmons Citrate Agar (SCA) and Pfizer Selective *Enterococcous* Agar (EA) (Huankai Microbial Technology Co., Ltd., Guangzhou, China), and incubated at 37 °C for 18–24 h. Typical *E. coli* colonies (large, blue-black and green metallic sheen) on EMB, *K. pneumoniae* colonies (the agar turns to blue) on SCA, *Enterococcus* spp. colonies (brown-black colony with brown-black halo) on EA and other colonies on EMB were streaked onto Soybean Casein Digest Agar (TSA, Huankai Microbial Technology Co., Ltd., Guangzhou, China). Isolates were purified using standard methods and grown in Tryptic Soy Polymyxin Broth Base (TSB; Huankai Microbial Technology Co., Ltd., Guangzhou, China) at 37 °C. After Gram-staining, Matrix-Assisted Laser Desorption/Ionisation Time of Flight Mass Spectrometry (MALDI-TOF-MS/ Autoflex speed TOF/TOF, Bruker, Germany) [65] was used for identification. The 166 *E. coli*, 68 *Enterobacter* spp.*,* 116 *K. pneumoniae* and 117 *Enterococcus* spp. isolates were stored in TSB containing 25% glycerol at − 80 °C.

### Antimicrobial susceptibility testing

Susceptibility to antimicrobials was determined in triplicate using the standard agar dilution method recommended by the Clinical and Laboratory Standards Institute (CLSI, 2020) [66]. The following eighteen antimicrobials were tested: kanamycin (KAN), gentamicin (GEN), erythromycin (ERY), azithromycin (AZM), norfloxacin (NOR), ofloxacin (OFX), ciprofloxacin (CIP), lomefloxacin (LOM), levofloxacin (LEV), sulfadiazine (SD), trimethoprim (TMP), ceftriaxone (CRO), cefixime (CFM), ampicillin (AMP), amoxicillin (AML), aztreonam (ATM), imipenem (IPM) and tetracycline (TET) (Meilun Biotechnology Co., LTD, Dalian, China). The isolates were grown on TSA plates, suspended in stroke-physiological saline solution to a turbidity equivalent to 0.5 McFarland Standard, and inoculated onto Mueller-Hinton agar plates using a multipoint inoculator (MIT-60P; Sakuma Seisakusyo, Tokyo, Japan). The final inoculum was approximately 10^4^ CFU per spot. Plates were incubated at 37 °C for 16–18 h. The range of 2-fold concentrations used to determine the susceptibility was determined by CLSI criteria. In addition, the results were interpreted in accordance with CLSI criteria (Supplementary Table S3). *E. coli* ATCC25922 and *E. faecalis* ATCC29212 were used as quality control strains.

### Detection of antimicrobial resistance genes

DNA was extracted by suspending an overnight culture grown on TSA in 600 μl of reagent-grade water, incubating the suspension at 100 °C for 10 min, centrifuging at 1100 g for 5 min and collecting the supernatant. The concentration and purity of the extracted DNA was estimated with a NanoDROP ONE (Thermo Scientific, USA) and a Qubit3.0 system (Life Invitrogen, USA). DNA extracts were stored at − 20 °C. Antibiotic resistance genes were amplified using primers and amplification conditions as described previously [2, 20, 22, 67–73] (Supplementary Table S4). Amplification products were assessed using electrophoresis in 1.0% (w/v) agarose gel. All results were confirmed by at least two independent experiments. Confirming that the amplification products were the target resistance genes was done using Sanger sequencing.

### Whole-genome sequencing of *K. pneumoniae*

Genomic DNA of *K. pneumoniae* K85 was extracted using an UltraClean1 Microbial DNA Isolation Kit (MoBio Laboratories, Inc., Carlsbad, CA, USA). The concentration and purity of the extracted DNA was estimated as described above. The genome of *K. pneumoniae* K85 was sequenced using Illumina NovaSeq PE150 at the Beijing Novogene Bioinformatics Technology Co., Ltd. (Beijing, China). The Raw data was filtered to obtain valid data (Clean Data). The sequences were assembled using SOAPdenovo (version 2.04) [74, 75], SPAdes [75] and ABySS [76], the assemblies were integrated with CISA [77] with default parameters. Then filling the gaps of preliminary assembly results, fragments below 500 bp were filtered out and the final result was counted for gene prediction. Antimicrobial resistance genes were predicted using the Comprehensive Antibiotic Research Database (CARD, https://card.mcmaster.ca) with default BLAST expectation value ≤ e^− 30^ and annotated with the highest score (default identity ≥40%, coverage ≥40%). The sequences were compared using BLASTN (https://blast.ncbi.nlm.nih.gov/Blast.cgi) and EasyFig 2.2.7 with default parameters [78]. Plasmid type analysis was done using database Enterobacteriales in Plasmid Finder v.2.0 with 100% identity and 60% coverage (https://cge.cbs.dtu.dk/services/PlasmidFinder/) [79].

### Data analysis

Statistical testing of the differences was tested using χ^2^ test of independence or Fisher’s exact test in IBM SPSS Statistics 26 software with default parameters [20]. A *P*-value < 0.05 was considered statistically significant. Other statistical analyses were done using Microsoft Excel (Microsoft, Inc., Washington DC, USA).

## Supplementary Information

**Additional file 1: Figure S1.** The proportion of antimicrobial resistant isolates from female and male giant pandas. KAN, kanamycin; GEN, gentamicin; AZM, azithromycin; ERY, erythromycin; NOR, norfloxacin; OFX, ofloxacin; CIP, ciprofloxacin; LOM, lomefloxacin; LEV, levofloxacin; SD, sulfadiazine; TMP, trimethoprim; CRO, ceftriaxone; CFX, cefixime; AMP, ampicillin; AML, amoxicillin; ATM, aztreonam; IPM, imipenem; TET, tetracycline.

**Additional file 2: Table S1.** Antimicrobial resistance profiles of the 144 *E*. *coli*, 66 *Enterobacter* spp., 110 *K. pneumoniae* and 43 *Enterococcus* spp. isolates from the feces of giant pandas in China. **Table S2** The location, sex and age of the sampled giant pandas. **Table S3** MIC breakpoints for Enterobacterales and *Enterococcus* spp. according to the Clinical and Laboratory Standards Institute (CLSI) guidelines. **Table S4** Primers, expected product sizes and annealing temperatures in the amplification of antimicrobial resistance genes.

## Data Availability

All data generated and analyzed in this study are included in this published article and the supplementary materials. The assembly genome sequencing data for *K. pneumoniae* strain K88 were deposited at CNGB Sequence Archive (CNSA) of China National GeneBank DataBase (CNGBdb) with accession number: CNP0002458 (https://db.cngb.org/cnsa/project/page/sub026616).

## Abbreviations

GPs: Giant pandas
AMR: Antimicrobial resistance
ARGs: Antibiotic resistance genes
MDR: Multidrug-resistant
ESBLs: Extended-spectrum β-lactamases
BPW: Buffered peptone water
MAC: MacConkey agar
EMB: Eosin methylene blue agar
SCA: Simmons Citrate Agar
EA: Pfizer Selective *Enterococcus* Agar
TSA: Soybean Casein Digest Agar
TSB: Tryptic Soy Polymyxin Broth Base
MALDI-TOF-MS: Matrix-Assisted Laser Desorption/Ionization Time of Flight Mass Spectrometry
KAN: Kanamycin
GEN: Gentamicin
ERY: Erythromycin
AZM: Azithromycin
NOR: Norfloxacin
OFX: Ofloxacin
CIP: Ciprofloxacin
LOM: Lomefloxacin
LEV: Levofloxacin
SD: Sulfadiazine
TMP: Trimethoprim
CRO: Ceftriaxone
CFX: Cefixime
AMP: Ampicillin
AML: Amoxicillin
ATM: Aztreonam
IPM: Imipenem
TET: Tetracycline
CLSI: The Clinical and Laboratory Standards Institute
CARD: The Comprehensive Antibiotic Research Database
