# Supplementary material for: Antimicrobial resistance of Escherichia coli, Enterobacter spp., Klebsiella pneumoniae and Enterococcus spp. isolated from the feces of giant panda
Source: BMC Microbiol. 2022 Apr 14;22:102. doi: 10.1186/s12866-022-02514-0 (PMC9008915; doi:10.1186/s12866-022-02514-0)
Supplement: Supplementary file 1 — Additional file 1: Figure S1. The proportion of antimicrobial resistant isolates from female and male giant pandas. KAN, kanamycin; GEN, gentamicin; AZM, azithromycin; ERY, erythromycin; NOR, norfloxacin; OFX, ofloxacin; CIP, ciprofloxacin; LOM, lomefloxacin; LEV, levofloxacin; SD, sulfadiazine; TMP, trimethoprim; CRO, ceftriaxone; CFX, cefixime; AMP, ampicillin; AML, amoxicillin; ATM, aztreonam; IPM, imipenem; TET, tetracycline. [file 12866_2022_2514_MOESM1_ESM.docx]

Figure S1: The proportion of antimicrobial resistant isolates from female and male giant pandas.

KAN, kanamycin; GEN, gentamicin; AZM, azithromycin; ERY, erythromycin; NOR, norfloxacin; OFX, ofloxacin; CIP, ciprofloxacin; LOM, lomefloxacin; LEV, levofloxacin; SD, sulfadiazine; TMP, trimethoprim; CRO, ceftriaxone; CFX, cefixime; AMP, ampicillin; AML, amoxicillin; ATM, aztreonam; IPM, imipenem; TET, tetracycline.
