# Supplementary material for: Antimicrobial resistance of Escherichia coli, Enterobacter spp., Klebsiella pneumoniae and Enterococcus spp. isolated from the feces of giant panda
Source: BMC Microbiol. 2022 Apr 14;22:102. doi: 10.1186/s12866-022-02514-0 (PMC9008915; doi:10.1186/s12866-022-02514-0)
Supplement: Supplementary file 2 — Additional file 2: Table S1. Antimicrobial resistance profiles of the 144 E. coli, 66 Enterobacter spp., 110 K. pneumoniae and 43 Enterococcus spp. isolates from the feces of giant pandas in China. Table S2 The location, sex and age of the sampled giant pandas. Table S3 MIC breakpoints for Enterobacterales and Enterococcus spp. according to the Clinical and Laboratory Standards Institute (CLSI) guidelines. Table S4 Primers, expected product sizes and annealing temperatures in the amplification of antimicrobial resistance genes. [file 12866_2022_2514_MOESM2_ESM.docx]

**Additional file 2**Table S1 Antimicrobial resistance profiles of the 144 *E*. *coli*, 66 *Enterobacter* spp., 110 *K.* *pneumoniae* and 43 *Enterococcus* spp. isolates from the feces of giant pandas in China.

| Resistance profile | *E. coli* (N) | *Enterobacter* (N) | *K. pneumoniae* (N) | *Enterococcus* (N) |
| --- | --- | --- | --- | --- |
| AML | 3 | 2 | 11 |  |
| AML/TET |  | 1 | 1 |  |
| AMP | 1 | 1 |  | 2 |
| AMP/AML | 6 | 39 | 40 |  |
| AMP/AML/ATM |  |  | 1 |  |
| AMP/AML/TET |  | 4 | 2 |  |
| AZM/AMP/AML |  | 1 |  |  |
| AZM/CIP/LOM/SD/TMP/CRO/AMP/AML/TET |  |  | 1 |  |
| AZM/SD | 1 |  |  |  |
| AZM/SD/AML/TET | 1 |  |  |  |
| AZM/SD/AMP/AML | 1 |  |  |  |
| CIP/SD | 1 |  |  |  |
| CRO/AMP/AML |  |  | 1 |  |
| ERY |  |  |  | 5 |
| ERY/AMP |  |  |  | 2 |
| ERY/AMP/TET |  |  |  | 3 |
| ERY/TET |  |  |  | 13 |
| GEN |  | 1 |  |  |
| GEN/AML |  | 2 |  |  |
| GEN/AMP/AML |  | 5 |  |  |
| GEN/CRO/CFM/AMP/AML/ATM/TET | 1 |  |  |  |
| KAN/AZM/SD/TMP/AMP/AML/TET |  | 1 |  |  |
| KAN/GEN/AZM/LEV/SD/CRO/CFM/AMP/AML/ATM/TET | 1 |  |  |  |
| KAN/GEN/AZM/LEV/SD/TMP/CRO/CFM/AMP/AML/ATM/TET | 1 |  |  |  |
| KAN/GEN/AZM/NOR/OFX/CIP/LOM/LEV/SD/TMP/CRO/CFM/AMP/AML/ATM/TET |  |  | 1 |  |
| KAN/GEN/SD/TET | 1 |  |  |  |
| KAN/SD/TMP/AMP/AML/TET | 1 |  |  |  |
| LEV/SD/TMP/AMP/AML/TET | 1 |  |  |  |
| NOR/LOM/LEV | 2 |  |  |  |
| NOR/OFX/CIP/LOM/LEV/SD/AMP/AML |  |  | 1 |  |
| NOR/OFX/CIP/LOM/LEV/SD/TMP/AMP/AML/TET | 2 |  |  |  |
| SD | 58 |  | 9 |  |
| SD/AML |  |  | 1 |  |
| SD/AML/TET |  |  | 1 |  |
| SD/AMP/AML | 5 | 2 | 25 |  |
| SD/AMP/AML/TET | 10 | 1 | 6 |  |
| SD/AMP/TET |  | 1 |  |  |
| SD/CRO/AMP/AML | 1 |  |  |  |
| SD/TET | 22 | 1 |  |  |
| SD/TMP/AML/TET | 2 |  |  |  |
| SD/TMP/AMP | 1 |  |  |  |
| SD/TMP/AMP/AML/TET | 2 | 1 | 7 |  |
| SD/TMP/AMP/TET | 3 |  |  |  |
| SD/TMP/CRO/CFM/AMP/AML/TET |  | 1 |  |  |
| SD/TMP/CRO/CFM/TET | 1 |  |  |  |
| SD/TMP/TET | 4 |  | 2 |  |
| TET | 10 | 2 |  | 18 |
| TMP/AMP/AML | 1 |  |  |  |

N, number of isolates. KAN, kanamycin; GEN, gentamicin; AZM, azithromycin; ERY, erythromycin; NOR, norfloxacin; OFX, ofloxacin; CIP, ciprofloxacin; LOM, lomefloxacin; LEV, levofloxacin; SD, sulfadiazine; TMP, trimethoprim; CRO, ceftriaxone; CFX, cefixime; AMP, ampicillin; AML, amoxicillin; ATM, aztreonam; IPM, imipenem; TET, tetracycline.

Table S2 The location, sex and age of the sampled giant pandas.

| Captive area | Sex | Age | Number |
| --- | --- | --- | --- |
| Huaying Mountain in Giant Panda Wild Training Base | Male | Adult | 2 |
| Nanjing Hongshan Forest Zoo | Male | Adolescent | 1 |
|  | Female | Adolescent | 2 |
| Strait (Fuzhou) Giant Panda Research and Exchange Center | Male | Adult | 1 |
|  | Female | Old | 1 |
| Langzhong Panda Park Science Museum | Male | Adult | 1 |
|  | Female | Adult | 1 |
| Tai'an Huahai Zoo | Male | Adult | 1 |
| Ningbo Youngor Zoo | Female | Adolescent | 2 |
| Tianmu Hunan Mountain Bamboo Sea Tourism | Female | Adult | 2 |
| Shijiazhuang Zoo Management Office | Male | Adult | 1 |
| Dafeng Port Zoo | Male | Adult | 2 |
| Dalian Forest Zoo | Male | Adult | 1 |
|  | Female | Adult | 2 |
| Meishan Qingshen China Bamboo Art City | Male | Adolescent | 1 |
|  | Male | Adult | 1 |
| Shennongjia Wildlife Theme Park | Male | Adult | 1 |
|  | Female | Adult | 1 |
| Suzhou Taihu Wetland World | Female | Adult | 2 |
| Shenyang Forest Zoo | Male | Adolescent | 1 |
|  | Female | Adolescent | 3 |
| Changyu Dongtian Panda Paradise | Male | Adult | 1 |
| Nanchang Zoo | Male | Adult | 1 |
| Baoding Aibao Wildlife World | Male | Adolescent | 2 |
|  | Female | Adolescent | 2 |
| Deqing County Rare Wild Animal Breeding Research Center | Male | Adult | 1 |
|  | Female | Adult | 1 |
| Shandong Linyi Zoological and Botanical Garden | Female | Adolescent | 1 |
|  | Male | Adult | 1 |
| Shanghai Wildlife Park | Male | Adolescent | 1 |
|  | Female | Adolescent | 1 |
|  | Female | Adult | 3 |
| Hangzhou Wildlife World | Female | Adult | 1 |
| Tianjin Zoo | Female | Adult | 1 |
| Chimelong Safari Park | Male | Adolescent | 5 |
|  | Female | Adolescent | 1 |
|  | Male | Adult | 3 |
|  | Female | Adult | 2 |
| Changchun Siberian Tiger Park | Female | Adolescent | 1 |
| Hetaoping, Bifengxia and Dujiangyan Base of China Giant Panda Conservation and Research Center | Male | Infant | 3 |
|  | Female | Infant | 5 |
|  | Male | Adolescent | 12 |
|  | Female | Adolescent | 11 |
|  | Male | Adult | 26 |
|  | Female | Adult | 43 |
|  | Male | Old | 2 |
|  | Female | Old | 5 |

Table S3 MIC breakpoints for Enterobacterales and *Enterococcus* spp. according to the Clinical and Laboratory Standards Institute (CLSI) guidelines.

| Antimicrobial agent | MIC Breakpoint, µg/mL (Enterobacterales) | | | MIC Breakpoint, µg/mL *(Enterococcus* spp.) | | |
| --- | --- | --- | --- | --- | --- | --- |
|  | S | I | R | S | I | R |
| Kanamycin（KAN） | ≤16 | 32 | ≥64 | - | - | - |
| Gentamicin（GEN） | ≤4 | 8 | ≥16 | - | - | - |
| Erythromycin（ERY） | - | - | - | 0.5 | 1-4 | 8 |
| Azithromycin（AZM） | ≤16 | - | ≥32 | - | - | - |
| Norfloxacin（NOR） | ≤4 | 8 | ≥16 | ≤4 | 8 | ≥16 |
| Ofloxacin（OFX） | ≤2 | 4 | ≥8 | - | - | - |
| Ciprofloxacin（CIP） | ≤0.25 | 0.5 | ≥1 | ≤1 | 2 | ≥4 |
| Lomefloxacin（LOM） | ≤2 | 4 | ≥8 | - | - | - |
| Levofloxacin（LEV） | ≤0.5 | 1 | ≥2 | ≤2 | 4 | ≥8 |
| Sulfonamides（SD） | ≤256 | - | ≥512 | - | - | - |
| Trimethoprim（TMP) | ≤8 | - | ≥16 | - | - | - |
| Ceftriaxone（CRO） | ≤1 | 2 | ≥4 | - | - | - |
| Cefixime（CFM） | ≤1 | 2 | ≥4 | - | - | - |
| Ampicillin（AMP） | ≤8 | 16 | ≥32 | ≤8 | - | ≥16 |
| Amoxicillin ^a^（AML） | ≤8 | 16 | ≥32 | - | - | - |
| Aztreonam（ATM） | ≤4 | 8 | ≥16 | - | - | - |
| Imipenem（IPM） | ≤1 | 2 | ≥4 | - | - | - |
| Tetracycline（TET） | ≤4 | 8 | ≥16 | ≤4 | 8 | ≥16 |

–, No CLSI breakpoints were available.

a. The breakpoint of amoxicillin testing was predicted by ampicillin.

Table S4 Primers, expected product sizes and annealing temperatures in the amplification of antimicrobial resistance genes.

| Antimicrobials class | Target gene | Primer sequence (5'→3') | Product size (bp) | Annealing temperature | Reference |  |
| --- | --- | --- | --- | --- | --- | --- |
| Aminoglycosides | *aph (3')-IIa* | TGACTGGGCACAACAGACAA | 677 | 52 | ([Zhang *et al.*, 2009](#_ENREF_60)) |  |
|  |  | CGGCGATACCGTAAAGCAC |  |  |  |  |
|  | *acc (3)-IIa* | ACCCTACGAGGAGACTCTGAATG | 384 | 52 |  |  |
|  |  | CCAAGCATCGGCATCTCATA |  |  |  |  |
|  | *acc (6')-Ib* | ATGACCTTGCGATGCTCTATGA | 486 | 55 |  |  |
|  |  | CGAATGCCTGGCGTGTTT |  |  |  |  |
|  | *ant (3'')-Ia* | ATCTGGCTATCTTGCTGACA | 284 | 50 |  |  |
|  |  | TATGACGGGCTGATACTGG |  |  |  |  |
| Macrolides | *ermE* | GAAAAGAGTACTCAACCAAATA | 616 |  |  |  |
|  |  | AGTAACGGTACTTAAATTGTTTA |  |  |  |  |
| Quinolones | *qnrA* | TCAGCAAGAGGATTTCTCA | 726 |  | ([Zou *et al.*, 2018](#_ENREF_64)) |  |
|  |  | GGCAGCACTATTACTCCCA |  |  |  |  |
|  | *qnrB* | CGACCTGAGCGGCACTGAAT | 515 | 57.5 |  |  |
|  |  | TGAGCAACGATGCCTGGTAG |  |  |  |  |
| Sulfonamides | *sul 1* | CATTGCCTGGTTGCTTCAT | 238 |  | ([Zhang *et al.*, 2009](#_ENREF_60)) |  |
|  |  | ATCCGACTCGCAGCATTT |  |  |  |  |
|  | *sul 2* | CATCATTTTCGGCATCGTC | 793 |  |  |  |
|  |  | TCTTGCGGTTTCTTTCAGC |  |  |  |  |
|  | *sul 3* | AGATGTGATTGATTTGGGAGC | 443 |  |  |  |
|  |  | TAGTTGTTTCTGGATTAGAGCCT |  |  |  |  |
| β-Lactams | *bla*_TEM_ | ATGAGTATTCAACATTTCCGTG | 840 |  | ([Essack *et al.*, 2001](#_ENREF_18)) |  |
|  |  | TTACCAATGCTTAATCAGTGAG |  |  |  |  |
|  | *bla*_SHV_ | TGGTTATGCGTTATATTCGCC | 1051 |  | ([Kim *et al.*, 1998](#_ENREF_28)) |  |
|  |  | GCTTAGCGTTGCCAGTGCT |  |  |  |  |
|  | *bla*_CTX-M_ | TTTGCGATGTGCAGTACCAGTAA | 544 |  | ([Edelstein *et al.*, 2003](#_ENREF_16)) |  |
|  |  | CGATATCGTTGGTGGTGCCATA |  |  |  |  |
|  | *bla*_IPM_ | CTACCGCAGCAGAGTCTTTG | 587 |  | ([Senda *et al.*, 1996](#_ENREF_48)) |  |
|  |  | AACCAGTTTTGCCTTACCAT |  |  |  |  |
|  | *bla*_VIM_ | AGTGGTGAGTATCCGACAG | 261 |  | ([Tsakris *et al.*, 2000](#_ENREF_52)) |  |
|  |  | ATGAAAGTGCGTGGAGAC |  |  |  |  |
| Tetracyclines | *tetA* | GGCACCGAATGCGTATGAT | 480 |  | ([Guo *et al.*, 2015](#_ENREF_19)) |  |
|  |  | AAGCGAGCGGGTTGAGAG |  |  |  |  |
|  | *tetB* | CTCAGTATTCCAAGCCTTTC | 416 |  |  |  |
|  |  | CTAAGCACTTGTCTCCTGTT |  |  |  |  |
|  | *tetC* | CTGGGCTGCTTCCTAATGC | 580 |  |  |  |
|  |  | AGCTGTCCCTGATGGTCGT |  |  |  |  |
|  | *tetM* | GTGGACAAAGGTACAACGAG | 406 |  | ([Ng *et al.*, 2001](#_ENREF_36)) |  |
|  |  | CGGTAAAGTTCGTCACACAC |  |  |  |  |
|  | *tetL* | GAAAAGAGTACTCAACCAAATA | 267 |  |  |  |
|  |  | AGTAACGGTACTTAAATTGTTTA |  |  |  |  |
